# Isolation of toxigenic *Corynebacterium diphtheriae* from cutaneous lesions in a donkey in Ontario, Canada, 2024: Implications for zoonotic disease transmission and One Health approach

**DOI:** 10.14745/ccdr.v52i0102a02

**Published:** 2026-02-19

**Authors:** Chidubem Okechukwu, Steven Rebellato, Heidi Pitfield, Kelly Magnusson, Ramien Sereshk, Durda Slavic, Heather McClinchey, Sarah Wilson, Julianne Kus, Colin Lee

**Affiliations:** 1 Public Health and Preventive Medicine, Northern Ontario School of Medicine University, Sudbury, ON; 2 Simcoe Muskoka District Health Unit, Barrie, ON; 3 Animal Health Laboratory, University of Guelph, Guelph, ON; 4 Ontario Ministry of Health, Toronto, ON; 5 Public Health Ontario, Toronto, ON; 6 Dalla Lana School of Public Health, University of Toronto, Toronto, ON; 7 Department of Laboratory Medicine and Pathobiology, University of Toronto, Toronto, ON

**Keywords:** diphtheria, *Corynebacterium diphtheriae*, Ontario, toxigenic, donkey, surveillance, One Health

## Abstract

This rapid communication describes a case of cutaneous lesions in a donkey in Ontario, Canada, from which toxigenic *Corynebacterium diphtheriae* (*C. diphtheriae*) was isolated. Seven human close contacts were identified and assessed. This communication focuses on public health challenges, interagency response and implications for One Health initiatives to prevent zoonotic transmission. Furthermore, it underscores the importance, successes and challenges of interagency collaboration to coordinate timely laboratory investigation, reporting, contact tracing, potential post-exposure prophylaxis and public education in responding to zoonotic disease. This investigation demonstrates the need for enhanced surveillance, clear legislative authority to facilitate reporting, and more specific guidance for close contact management of *C. diphtheriae* and other zoonotic agents in animals, which can cause morbidity and mortality in humans.

## Introduction

*Corynebacterium diphtheriae* (*C. diphtheriae*), the primary agent of diphtheria, is an aerobic or facultatively anaerobic, Gram-positive, rod-shaped bacterium; some strains carry the diphtheria tox gene that produces a potent toxin that causes severe disease in humans ((1)). Other *Corynebacterium* species that can acquire the diphtheria toxin gene are *Corynebacterium ulcerans* and *Corynebacterium pseudotuberculosis* ((2)). *Corynebacterium* are very common in the environment, including soil, plants, animals and humans ((1)); however, toxigenic *C. diphtheriae* is rare and typically associated with human infection, although domestic animals such as cats, dogs, and horses have been identified as carriers of this organism ((1)). The incubation period for human *C. diphtheriae* infection ranges from 2–10 days; the mode of transmission to humans is direct contact for cutaneous lesions and via droplet for respiratory diphtheria ((3,4)). This case highlights a rare instance of toxigenic *C. diphtheriae* isolated from a domestic animal, underscoring the need for vigilance in zoonotic surveillance and cross-sectoral collaboration to mitigate public health risks.

*Corynebacterium diphtheriae* is of great public health significance due to its capacity to produce a potent toxin that can lead to severe complications such as myocarditis, kidney failure and death ((5)). Without prompt treatment, the case fatality rate for diphtheria ranges from 5% to 10%, with higher mortality rates (up to 30%) observed among unvaccinated individuals, particularly children under five years old and adults over 40 years of age ((6,7)). Before the introduction of the diphtheria toxoid vaccine in the 1920s, the disease caused approximately 100,000–200,000 cases and 13,000–15,000 deaths annually in the United States ((8)). In Canada, routine immunization has significantly reduced the incidence of diphtheria; however, 19 cases were reported between 1993 and 2012, with the most recent fatalities occurring in a Canadian resident in 2010 and a visitor to Canada in 2018 ((9,10)). Globally, diphtheria remains endemic in regions with low immunization coverage. In addition, displacement of populations due to political or economic instability, combined with disruptions in immunization infrastructure, has contributed to recent outbreaks in several countries, including among asylum seekers in Europe ((5)). Furthermore, a serosurvey of young healthy adult Canadians noted that approximately 20% of individuals do not have adequate levels of antibodies for diphtheria ((11)). The re-emergence of diphtheria underscores the critical importance of maintaining high vaccination coverage and robust public health systems to prevent the spread of this potentially fatal disease.

Based on available literature, this is the first documented case of toxigenic *C. diphtheriae* in a donkey. Previous reports have identified *C. diphtheriae* in various animals, including dogs, cats, horses, a cow and a fox, but only isolates from two dogs and two horses were confirmed to be toxigenic ((12)). To date, there are no confirmed cases of zoonotic transmission of toxigenic *C. diphtheriae* to humans. A 2022 case involving a toxigenic strain in a pet cat in Texas did not result in human infection; however, zoonotic transmission has been well documented with toxigenic *C. ulcerans*, a related species capable of producing diphtheria toxin, particularly from domestic dogs and cats to humans ((13–16)).

In September 2024, the Animal Health Laboratory (AHL) based in Guelph, Ontario reported to the Ontario Ministry of Health about the isolation of a potentially toxigenic *C. diphtheriae* isolate from a donkey with cutaneous lesions. Subsequently, the relevant local public health unit in Ontario was notified. In Ontario, toxin-producing *C. diphtheriae* in humans is a pathogen of public health significance that requires reporting to public health authorities, as there are interventions that can be implemented to prevent further transmission, such as the identification of asymptomatic carriage among close contacts and offering post-exposure chemoprophylaxis (antibiotics) and immunoprophylaxis (vaccine) ((17)). There is also a potential risk of transmission of toxigenic *C. diphtheriae* from animals or laboratory isolates to humans, necessitating human contact management that may include post-exposure antibiotic and vaccine advice, following a risk assessment ((18,19)). In animals, toxigenic *C. diphtheriae* bacteria are not designated as immediately notifiable in Ontario by laboratories or veterinarians to the Ontario Ministry of Agriculture, Food and Agribusiness (OMAFA) and/or to the Public Health Unit/Ministry of Health, which led to challenges for public health surveillance and timely response when a toxigenic case was identified, especially as this was the first known animal case of toxigenic *C. diphtheriae* in Ontario.

This report highlights the zoonotic risk of toxigenic *C. diphtheriae* isolated from animals and the challenges in public health surveillance and management due to the absence of legislative clarity for animal-to-human transmission. This case emphasizes the critical need for a One Health approach that integrates animal and human health systems to prevent zoonotic transmission and enhance response coordination. Hence, the objective of this report is to describe the epidemiological, diagnostic, and public health management of isolation of toxigenic *C. diphtheriae* from a donkey, highlighting the implications for zoonotic transmission and underscoring the need for policy enhancements to support timely outbreak response and public health safety.

## Current situation

### Case presentation

**Figure 1:**
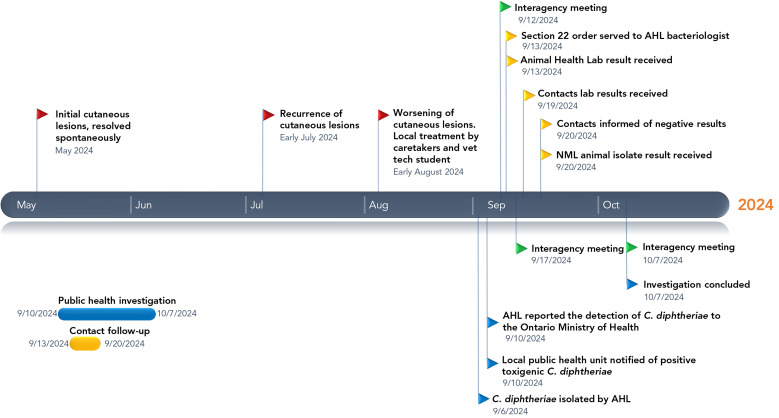
Timeline of events in the investigation of a zoonotic cutaneous diphtheria case in a donkey, Ontario, Canada, 2024 Abbreviations: AHL, Animal Health Laboratory; *C.*, *Corynebacterium*; NML, National Microbiology Laboratory

A 35-year-old donkey was evaluated by a veterinarian for non-healing cutaneous lesions on all four limbs (**Figure 1**). The lesions (wounds) first appeared in May 2024, resolved spontaneously and recurred in July 2024. The wounds worsened in August 2024 necessitating examination by the attending veterinarian in early September 2024. The veterinarian assessed and treated the donkey at the owner’s property, collected samples from the wounds for bacterial culture and delivered treatment, including antibiotics and a topical cream application (corticosteroid with an antibiotic) on the wound surface.

### Laboratory findings

The swabs of the lesions grew a mixed culture of *C. diphtheriae*, as well as *Pseudomonas aeruginosa* and *Streptococcus equi* subspecies *zooepidemicus*. The former was positive for the toxin gene at the AHL by a research use only (RUO) PCR assay ((20)). Molecular detection of the diphtheria toxin gene is not routinely done at AHL given the rarity of *C. diphtheriae* isolation from animal specimens; however, a noticeable rise in referrals of isolates to the National Microbiology Lab (NML) for diphtheria toxin testing and recent reports of diphtheria toxin gene carrying *C. ulcerans* isolates prompted the lab to keep the primers and a probe in stock ((21)). Despite the lack of real-time PCR (qPCR) verification at AHL, due to the high specificity of the published test, there was confidence in the high probability (approximately 92%) that this was a true positive.

The isolate was sent to NML for qPCR confirmation and for detection of toxin production by modified Elek test. At NML, the identification was confirmed to be *C. diphtheriae*, which was positive by both the PCR for the diphtheria toxin gene and the modified Elek test for the phenotypic detection of the expressed diphtheria toxin.

### Public health notifications and challenges

When the toxigenic *C. diphtheriae* isolate was identified at AHL, the potential risk to human health was recognized and this finding was reported to the Ontario Ministry of Health.

There is no supporting legislation or policy to support the release of personal information to any of the relevant provincial and local public health agencies, which is of critical importance for outbreak tracing and other public health measures. *Corynebacterium diphtheriae* in animals is not designated as a provincially notifiable hazard to OMAFA ((22)). Similarly, the *Health Protection and Promotion Act, R.S.O. 1990, c.H.7*, does not require confirmation of *C. diphtheriae* in animals to be reported to the Ontario Ministry of Health or the local public health agency. This led to a discussion among the AHL, OMAFA and the provincial and local public health authorities on how to proceed with the animal case and with human contact management while respecting both the legislation and the privacy of the donkey owner, including their location and caretaker identification. Eventually, it was agreed that, at that time, the best way forward was for the local medical officer of health to issue a section 22 order regarding a communicable disease under the *Health Protection and Promotion Act*. The order was issued to the AHL veterinary bacteriologist requiring the contact details and laboratory reports to be provided to the Medical Officer of Health to enable the local public health unit to conduct a public health investigation and potentially institute adequate control measures, such as further laboratory testing, and, where necessary, chemoprophylaxis and immunization of close human contacts.

The animal’s history and clinical presentation were crucial in guiding the diagnostic approach and subsequent public health contact management. It is unclear how the donkey became infected with *C. diphtheriae*; however, the donkey’s age (35 years), likely immunosenescence with a greater propensity to acquire and develop the disease from possible human and/or animal carriers ((5)), the potential for underlying skin disease from seasonal biting insects and chronic exposure to soil where *C. diphtheriae* may have been present ((1)) may have created the perfect situation for the development of cutaneous lesions. Of note, the donkey spent most of the summer outdoors. The initial lesions in May 2024 could plausibly be linked to environmental exposure, such as contact with contaminated soil, which is consistent with known reservoirs of *C. diphtheriae*. The recurrence and worsening of the lesions in July and August 2024 may have been exacerbated by seasonal factors like biting insects and increased outdoor exposure.

Diphtheria is a disease of public health significance; hence, there were concerns regarding human contact. Notably, the donkey did not display respiratory symptoms, so transmission by droplet was not deemed a concern.

### Contact tracing and management

Close contacts were defined as persons who had close contact with the donkey and/or exposure to mucous membranes and/or provided direct care since the onset of symptoms in the animal. For the months during which the donkey had the cutaneous lesions, there were seven known human close contacts. The human contacts include two caretakers who provided regular care to the donkey, one veterinary technician student who provided direct wound care to the donkey when the caretakers travelled, a veterinarian who also provided direct care with gloves, took samples of lesions and submitted the wound culture, two individuals who reportedly had no direct contact with the wound but fed and held the chain of the donkey in close proximity at multiple times over the past two months, and one person who trimmed the donkey’s hooves. Animal contacts on the same property included one horse that lived in the same barn with the donkey in the winter months, and a dog.

Post-exposure *C. diphtheriae* contact management includes chemoprophylaxis with either one dose of intramuscular benzathine penicillin G or 7–10 days with a macrolide antibiotic, and diphtheria vaccination if the contact’s vaccine status is not up to date ((17,21)). Ontario’s Infectious Disease Protocol and the United States Centers for Disease Control and Prevention (CDC) provide advice for contacts of toxigenic *C. diphtheria* species occurring in humans and recommend vaccinating close contacts if their last diphtheria-containing vaccination was more than five years ago ((17,23)). On the other hand, the United Kingdom (UK) and Australia have guidance on human contact management when toxigenic *Corynebacterium* spp. are identified in animals, with the UK’s guidance document and Australian guidelines recommending a booster dose of diphtheria-containing vaccine if more than 12 months after the last dose ((24,25)). Following the precautionary principle, a dose of diphtheria-containing vaccine was offered to all close contacts if their last dose was more than 12 months ago.

### Outcomes and follow-up

All contacts had received diphtheria vaccination within the past decade, most within the last five years, as shown in **Table 1**.

**Table 1:** Post-exposure prophylaxis (PEP) provided to close contacts

Human contacts	Year received last dose of diphtheria-containing vaccine	Interventions
1	2019	Tdap Provided; took antibiotic PEP 5/7 days
2	2022	Tdap Provided; took antibiotic PEP 5/7 days
3	2022	Declined Tdap; did not take antibiotic PEP
4	2018	Tdap Provided; took antibiotic PEP 5/7 days
5	Unsure	Tdap Provided; did not take antibiotic PEP
6	2021	Tdap Provided; did not take antibiotic PEP
7	2019	Declined Tdap; did not take antibiotic PEP

Abbreviations: PEP, post-exposure prophylaxis; Tdap, tetanus, diphtheria, pertussis

Given the high probability that a PCR tox positive isolate would express the toxin ((26)), we elected to offer post-exposure prophylaxis, as per the UK and Australian guidelines on human exposure to animal *Corynebacterium* spp. ((24,25)), while we waited for confirmation of toxigenicity by the NML (turnaround time for testing was about one week). Post-exposure prophylaxis consisted of seven days of azithromycin 500 mg oral daily and a diphtheria-containing vaccine if the last dose was more than 12 months prior. In addition, health education on asymptomatic carriage, signs and symptoms to monitor, as well as proper hygiene practices, was provided. All contacts remained asymptomatic from the period of contact to the period of assessment, except for one contact who developed a sore throat two weeks after contact with the donkey. Nasopharyngeal swabs for *C. diphtheriae* culture were taken from all contacts to assess potential human transmission, and at the same time, antibiotic prophylaxis was offered, with three individuals accepting the antibiotics. No swabs yielded growth of *C. diphtheriae* on culture, and the contacts were informed of the negative laboratory results. Consequently, the three contacts who started the medication stopped on day 5/7, while the others who were awaiting the lab results before the start of the antibiotics did not start the medication. Follow-up with the donkey owner revealed that the donkey was doing better with only one wound that required dressing, and there was no further requirement for pain medications. Although the human contacts tested negative for *C. diphtheriae* carriage, given the positive modified Elek testing of the animal isolate, there was a potential ongoing risk of transmission from animal to human. Hence, the owner was advised to take precautions, particularly the use of personal protective equipment, when dealing with the donkey’s secretions. The contact investigation was a critical component of the public health response, requiring rapid identification and risk assessment of potentially exposed individuals.

## Recommendations

Legislative changes to strengthen reporting protocols for human contact management are needed to improve the surveillance and prevention of transmission of *C. diphtheriae* and other zoonotic infections. It is essential to establish and strengthen routine communication and collaboration between veterinary diagnostic laboratories and public health laboratories that process human specimens. This collaboration would allow for the timely submission of suspicious isolates from veterinary sources to public health laboratories for confirmation. Currently, this practice is not routinely implemented, and public health laboratories do not typically accept isolates from animal sources. As the global health landscape evolves, preparedness for re-emerging zoonotic diseases like diphtheria will remain a critical challenge for public health authorities; hence, the need to streamline reporting of diphtheria and other relevant diseases of public health significance in humans, through a lens of the One Health approach.

## Conclusion

Current findings establish that isolation of toxigenic *C. diphtheriae* from cutaneous lesions in animals, although rare, can carry zoonotic transmission risks, particularly through direct contact with open lesions. The investigation confirmed the presence of toxigenic *C. diphtheriae* in the affected donkey and highlighted the necessity for considering vigilant health measures, including close contact management and post-exposure prophylaxis for at-risk individuals. Precautionary post-exposure antibiotic treatment and vaccination were provided at the same time *C. diphtheriae* cultures were taken from the close contacts. The cultures did not grow *C. diphtheriae*. Nonetheless, this incident highlights the importance of maintaining up-to-date diphtheria booster vaccinations within the population. Despite the excellent interagency collaboration in this case, critical uncertainties remain, notably regarding the specific transmission dynamics of toxigenic *C. diphtheriae* from animals to humans and the potential for other domestic or wild animals to act as carriers. Additionally, the absence of standardized surveillance and mandatory reporting for toxigenic *C. diphtheriae* and other diphtheria toxin gene carrying *Corynebacterium* spp. cases in animals pose a significant gap, complicating timely public health responses.
